# Tumour Necrosis Factor Alpha, Interferon Gamma and Substance P Are Novel Modulators of Extrapituitary Prolactin Expression in Human Skin

**DOI:** 10.1371/journal.pone.0060819

**Published:** 2013-04-23

**Authors:** Ewan A. Langan, Silvia Vidali, Natascha Pigat, Wolfgang Funk, Erika Lisztes, Tamás Bíró, Vincent Goffin, Christopher E. M. Griffiths, Ralf Paus

**Affiliations:** 1 Dermatology Research Centre, Manchester Academic Health Science Centre, and Institute of Inflammation and Repair, University of Manchester, Manchester, United Kingdom; 2 Department of Dermatology, University of Lübeck, Lübeck, Germany; 3 Inserm U845/Centre de Recherche Croissance et Signalisation, Faculté de Médecine, Université Paris Descartes, Sorbonne Paris Cité, Paris, France; 4 Klinic Dr. Kozlowski, Munich, Germany; 5 DE-MTA “Lendület” Cellular Physiology Research Group, Department of Physiology, University of Debrecen, Debrecen, Hungary; Baylor College of Medicine, United States of America

## Abstract

Human scalp skin and hair follicles (HFs) are extra-pituitary sources of prolactin (PRL). However, the intracutaneous regulation of PRL remains poorly understood. Therefore we investigated whether well-recognized regulators of pituitary PRL expression, which also impact on human skin physiology and pathology, regulate expression of PRL and its receptor (PRLR) *in situ.* This was studied in serum-free organ cultures of microdissected human scalp HFs and skin, i.e. excluding pituitary, neural and vascular inputs. Prolactin expression was confirmed at the gene and protein level in human truncal skin, where its expression significantly increased (p = 0.049) during organ culture. There was, however, no evidence of PRL secretion into the culture medium as measured by ELISA. PRL immunoreactivity (IR) in female human epidermis was decreased by substance P (p = 0.009), while neither the classical pituitary PRL inhibitor, dopamine, nor corticotropin-releasing hormone significantly modulated PRL IR in HFs or skin respectively. Interferon (IFN) γ increased PRL IR in the epithelium of human HFs (p = 0.044) while tumour necrosis factor (TNF) α decreased both PRL and PRLR IR. This study identifies substance P, TNFα and IFNγ as novel modulators of PRL and PRLR expression in human skin, and suggests that intracutaneous PRL expression is not under dopaminergic control. Given the importance of PRL in human hair growth regulation and its possible role in the pathogenesis of several common skin diseases, targeting intracutaneous PRL production via these newly identified regulatory pathways may point towards novel therapeutic options for inflammatory dermatoses.

## Background

Whilst prolactin (PRL) is appreciated for its role in the modulation of hair growth, both in human and other mammalian species [1], [2], less attention has been afforded to the role(s) of PRL in cutaneous biology and pathology in general. However, several recent publications have reawakened interest in the “PRL-skin” connection, particularly in the context of a possible role for PRL in psoriasis [3], [4], [5], [6] and systemic lupus erythematosus [7].

However, in human skin, the published literature has only confirmed scalp skin and scalp hair follicles (HFs) as cutaneous sources of extra-pituitary PRL production [1], although PRL expression has also been reported in human dermal fibroblasts *in vitro* [8]. Also, El-Khateeb *et al.* [5] recently reported increased levels of PRL in blister fluid from lesional psoriatic skin in psoriasis patients when compared to uninvolved skin and skin from healthy subjects. Moreover, these levels exceeded serum PRL levels; evidence that PRL is produced intracutaneously. In contrast, Slominski *et al* failed to identify PRL gene expression in both normal and pathological skin [9], and Björntorp *et al* could not identify PRL gene expression in involved skin in psoriasis using reverse transcriptase polymerase chain reaction [10]. Given the pro-inflammatory cutaneous cytokine milieu which is present in psoriasis, we speculated that cytokines, for example tumour necrosis factor alpha (TNFα) and interferon gamma (IFNγ), may up-regulate intracutaneous PRL production.

Furthermore, although the regulation of pituitary PRL synthesis and release has been extensively studied [11], albeit almost exclusively in rodent models [12], much less is known about the regulation of extra-pituitary PRL production [13] (Table S1), namely in human skin. Given that the “regulation of” human “extra-pituitary PRL release can only be studied in human cells and tissues” [12], human skin and HFs provide an invaluable resource for studying the regulation of extra-pituitary PRL gene and protein expression. Conventionally, the regulation of extra-pituitary PRL synthesis and secretion was considered to differ from that in the pituitary, based on the assumption of dual promoter usage in extra-pituitary versus pituitary tissues, the latter involving the pituitary specific transcription factor Pit-1 [14], [15]. However, recent studies exploring the autocrine/paracrine actions of PRL [16] have recapitulated the pro-apoptotic effects of PRL observed in the HF [1], inviting the hypothesis that the HF itself can be utilized to study the regulation and autocrine/paracrine action of PRL in humans [17].

Given (i) the lack of any universal PRL stimulatory/inhibitory factor [18], (ii) that little is known about the regulation of PRL receptor (PRLR) expression in extra-pituitary sites [19], and (iii) that conclusions drawn from studies determining the regulation of PRL and PRLR expression in other sites cannot be reliably extrapolated to the skin, studies with human skin and HFs are best placed to determine the regulation of intracutaneous PRL and PRLR. Furthermore, the skin and HF organ culture model has already provided novel insights into the *in vitro* regulation of PRL and PRLR in human skin and offers unparalleled accessibility and clinical utility [20], [21]. Given the major endocrine functions of skin [22], [23], [24], [25], [26], a source and target of PRL [1], and that PRL is a potential player in skin and hair diseases [3], [4], [5], [6], [7], [27], [28], [29] a comprehensive analysis of the intracutaneous regulation of PRL and PRLR is required.

Therefore we determined whether healthy corporal human skin expresses PRL and PRLR expression at the gene and protein level, and establish whether there are any time-dependent changes in cutaneous PRL and PRLR expression in organ culture *in vitro.* Moreover, we asked whether selected regulators of pituitary PRL synthesis and/or secretion and pro-inflammatory cytokines alter epidermal and follicular PRL expression. Finally, we examined whether there are any differences between the regulation of PRLR expression in the skin and the HF.

## Materials and Methods

Human skin was obtained as by-products of cosmetic surgery, after written informed consent was provided. Full ethical approval was obtained from the University of Lübeck ethics committee, according to the Helsinki Declaration. For the determination of PRL and PRLR immunoreactivity (IR), immunohistochemistry was performed as described previously [20]. In summary, after drying at room temperature and fixing in acetone, sections were treated with 3% hydrogen peroxide to block any endogenous peroxidase. After washing in tris-buffered sulphate (TBS), sections were treated with an avidin and biotin blocking kit (Vector, Burlingame, CA) and then pre-incubated with 10% normal rabbit serum. Prolactin antibody (Prolactin (C-17) goat polyclonal antibody, raised against a peptide mapping near the C-terminus of prolactin of human origin, sc-7805, Santa Cruz, CA)[1] was then applied overnight at 4°C, 1∶50 dilution in TBS with 2% normal rabbit serum. Negative control was by the omission of the primary antibody and overnight incubation with 2% normal rabbit serum in TBS. After further washes with TBS, rabbit anti-goat biotinylated antibody, 1∶200 dilution in TBS with 2% normal rabbit serum, was applied for 45 minutes at room temperature. After further washes, the avidin-biotin peroxidase kit was used for 30 minutes at room temperature (Vector, Burlingame, CA). 3-amino-9-ethyl-carbazole (AEC) (Vector, Burlingame, CA) was then used as the chromogen. Sections were then counterstained with Haematoxylin and mounted with Faramount (Dako, Glostrup, DK).

For PRLR immunohistochemistry, PRLR antibody (mouse monoclonal PRLR antibody, reacts with the extracellular portion of the receptor, isotype IgG1, SM5033P, Acris antibodies, DE)[30], [31] was applied (1∶50) in antibody diluent (DCS Innovative Diagnostik-Systeme, Hamburg, DE) overnight at 4°C and the labelled streptavidin biotin (LSAB*)* (DCS Innovative Diagnostik-Systeme, Hamburg, DE) method was used. The application of antibody diluent alone served as the negative control. AEC (Vector, Burlingame, CA) was again applied as the chromogen, prior to counterstaining with haematoxylin and mounting with Faramount (Dako, Glostrup, DK).

Immunoreactivity for PRL and PRLR in the HF and sebaceous gland served as internal positive controls, based on previously published expression patterns [1], [20]. Four millimetre punch biopsies were obtained from redundant corporal skin. (for PRL immunohistochemistry, ♀:n = 3 Age: 28–57 years, for PRLR immunohistochemistry, ♀:n = 3 Age:28–63). The redundant skin from the 28 year old female was used in both PRL and PRLR analyses.

### Investigating the temporal regulation of PRL

In order to investigate the effect of organ culture itself on PRL and PRLR IR, 4 mm punch biopsies were obtained from redundant corporal skin, by-products of cosmetic surgery, (female, n = 3, age 42–63 years) and placed in serum free culture medium as described previously [20], [32]. Culture medium consisted of William’s E (Biochrome, Berlin DE), supplemented with 2 mmol/l L-glutamine (Invitrogen, Paisley, UK), 10 ng/ml hydrocortisone (Sigma-Aldrich), 10 µg/ml insulin (Sigma Aldrich) and antibiotic mixture (100 IU/ml penicillin and 10 µg/ml streptomycin) [32]. The medium was changed every 48 hours. After 7 days in culture, skin was either embedded in cryomatrix (Thermo Shandon, Cheshire, UK), snap frozen in liquid nitrogen, sectioned (7 µm) and stained for PRL/PRLR as above or directly frozen for quantitative “real-time” PCR (qRT-PCR) studies.

### Investigating the intracutaneous regulation of PRL

Microdissected female anagen VI HFs were isolated from redundant scalp skin as described [1]. Microdissected HFs and/or full thickness skin [32] were cultured in supplemented serum-free culture medium for either 24 hours or 7 days. For skin 24 hour skin organ culture, the following substances were tested. Substance P (Sub P, 100 nM, 2♀ subjects aged 45–46 years)[33], corticotropin releasing hormone (CRH, 1 µM, 3♀ subjects aged 45–57 years)[34], triiodothyronine (T3, 100 pM)[35] or thyroxine (T4, 100 nM) 3–5♀ aged 45–71 years) [35], [36].

To enable a comparison between the effects of PRL on PRLR protein expression in the skin and HF, 7 day organ culture with PRL (400 ng/ml) was used. For skin organ culture, skin from 3♀ subjects (aged 42–63 years), was compared with HFs from 4♀ subjects (aged 49–68 years). For the remaining HF studies, the following substances were used, dopamine (DA 10–1000 nM, 3♀ aged 47–68 years)[37], TNFα (0.5–50 ng/ml, 3♀ subjects aged 49–68 years)[38] and IFNγ (75 IU/ml, 3♀ subjects aged 44–68 years, added only at day 1 and 5) [39]. The concentration of IFNγ (75 IU/ml) was selected given that we have previously shown that

75 IU/ml IFNγ does not induce catagen, while higher doses would have greatly reduced the percentage of anagen HFs. [39], [40]. All HF experiments started with 12–18 HFs. At the end of each culture, only anagen VI HFs were used for analysis to avoid any confounding effects of hair cycle phase on PRL and PRLR expression [1]. The regulation of PRL on its own expression could not be determined since the skin and HF have endogenous PRL production [1], [20], [21], preventing differentiation of endogenous from exogenous PRL by immunohistochemistry. Cultured skin and HFs were then cryosectioned (HFs:6 µm, skin:7 µm) and stained for PRL and/or PRLR IR as described above.

For qRT-PCR analyses, skin and/or HFs were treated for either 24–48 hours or 7 days (see figure legends for details).

### Phosphorylated STAT5 studies

In order to determine whether PRLR-mediated signaling involved the canonical JAK2/STAT5 pathway, phosphorylated STAT5 levels were examined using immunohistochemistry, employing phospo-STAT5 (Tyr694) rabbit monoclonal antibody to detect STAT5a and b proteins when phosphorylated at Tyr 694 (Cell Signaling, C11C5; 1∶100 dilution). After fixation in acetone and blocking of endogenous peroxidase, blocking solution (1% horse serum in 3% BSA-TBS-Tween) was applied for 60 minutes, followed by overnight incubation with the primary antibody at 4°C. Horse anti-rabbit biotinylated secondary antibody (Vector lab BA-1100; 1∶200 dilution in TBS-T +2% horse serum) was then applied for 30 minutes at room temperature, followed by the Vectastain Elite ABC® (Vector lab, PK-6100) detection technique as described above, with 3,3' diaminobenzidine (DAB) used as the chromogen.

### Quantitative “real-time” PCR studies

Specific mRNA transcripts of were analyzed by qRT-PCR as described (40), using TaqMan primers and probes (PRL, Assay ID: Hs 00168730_m1; PRLR, Assay ID: Hs00168739_m1). As internal housekeeping gene controls for the qRT-PCR experiments, transcripts of glyceraldehyde 3-phosphate dehydrogenase (GAPDH, Assay ID: Hs 99999905_m1), β-actin (ACTB, Assay ID: Hs 99999903_m1), and cyclophilin A (PPIA, Assay ID: Hs99999904_m1) were determined. The amount of the aforementioned transcripts was normalised to those of the most stable control gene using the ΔΔCT method. All experiments were performed in triplicate. The expression of the target genes was normalized to that of the relevant control group and independent data from 2–3 different donors were pooled, and expressed as fold changes. Statistical analysis was performed by paired or unpaired two-sample Student’s t-test.

### ELISA studies

To determine whether there was any evidence of PRL secretion into the culture medium, ELISA was performed on conditioned culture media from two subjects from day one and day five. This media was from skin (♀ 31 years) and HF organ culture (♀ 52 years) respectively. ELISA was performed as described previously [41]. In brief, 100 µl of conditioned media was added to each well. After 1 hour of shaking at room temperature, subsequently washing with buffer, 100 µl of rabbit-anti human PRL primary detected antibody was added (Dako A0569 1∶2500). After 1 hour of shaking at room temperature, then washing, HRP-conjugated detection antibody (Cell signaling 7074, rabbit IgG, 1∶5000) was added for 1 hour at room temperature. After washing, TMB was added to each well for 15–20 minutes (in the dark), followed by stop solution (50 µl). The plate was then read at 450 nm within 20 minutes of the addition of the stop buffer. The lower limit of detection of PRL was 0.445 ng/ml.

### Outer root sheath keratinocyte cultures

Given that PRL and PRLR are prominently expressed in the outer root sheath (ORS) compartment of human HFs, human ORS keratinocyte cell culture was performed to verify whether PRL and PRLR transcripts are also expressed in isolated HF keratinocytes. Moreover, we checked the expression of PRL and PRLR after treatment with TNFα (5 ng/ml for 6 hours) and tested whether this treatment influenced expression of STAT5, a key molecule in PRLR mediated signaling. ORS keratinocytes were obtained from human HFs (2♀ subjects aged 25–26 years) after digestion with trypsin [42] and cultured in serum-free media [21].

### Statistical Analysis

Standardised reference areas in the ORS were measured for PRL and PRLR IR using National Institute of Health Image J software (Bethesda, Maryland, USA, See - Supplement 1) [20], [34], [43]. 2–6 measurements were taken for each anagen HF and the mean IR calculated. For epidermal immunohistomorpometric analyses, IR of the entire epidermis, excluding the stratum corneum, was measured in 3 high power fields (x200 magnification). For statistical analyses, data were checked for normal distribution by D’Agostino and Pearson normality test. Depending on the data distribution and the number of groups investigated, Student’s t-test/one way ANOVA (with Bonferroni’s post hoc test) or Mann-Whitney U-test/Kruskal-Wallis test (with Dunn’s multiple comparison post hoc test) were used where appropriate.

Statistical analysis was performed using GraphPad Prism, Version 5 (San Diego California, USA). p<0.05 was considered as statistically significant and results were expressed as the mean +/− standard error of the mean (SEM). To combine the results from the independent quantitative (immuno-) histomorphometry experiments, the results from the treatment groups were normalized to the control group (set at 100) and the results were pooled. Significance was determined as *p<0.05, **p<0.01, ***p<0.001.

## Results

### PRL immunoreactivity is present in corporal skin and PRL gene transcription can increase during human skin organ culture

**Figure 1:**
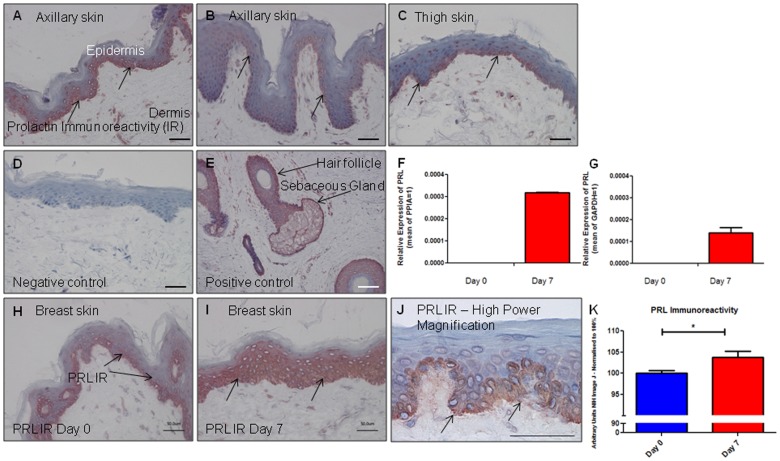
Prolactin immunoreactivity is present in corporal skin and its gene transcription can increase during culture. Prolactin (PRL) immunoreactivity (IR) is demonstrated in (A) axillary skin from a 49 year old ♀, (B) axillary skin from a 28 year old ♀ and (C) thigh skin from a 57 year old ♀. The black arrows show that the IR was most prominent in the basal layer of the epidermis, in a cytoplasmic distribution, in non-scalp skin. (D) Omission of the primary antibody served as the negative control and (E) the hair follicle outer root sheath keratinocytes and sebaceous gland served as internal positive controls. (F) PRL gene expression was below the level of detectability at day 0, but became detectable by day 7 during serum free organ culture in breast skin (from a 42 year old ♀) and (G) abdominal skin (from 63 year old ♀). At the protein expression level, PRL IR also increased between (H) day 0 and (I) day 7. (J) High magnification of PRL IR. Quantitative measurement of PRL IR, using Image J software is shown in (K). Results pooled from 3 females aged 42–63 years. Increased protein expression correlated with increased gene expression in two subjects over the same time period. However, in one subject PRL gene expression was readily detectable at day 0, but decreased significantly during organ culture (data not shown). qRT-PCR results could not be normalized, combined and expressed as fold change from day 0, as no PRL gene expression was detected at day 0 in 2/3 subjects. All scale bars represent 50 µm.

To determine whether PRL protein was expressed in corporal skin, PRL immunohistochemistry was performed. Prolactin specific staining was present in each subject, in a cytoplasmic distribution in the basal epidermal layer (Fig 1A–C). This was consistent with the previously described PRL distribution pattern in scalp skin, whose specificity had been established by RT-PCR analyses and by studying pituitary sections [20]. By qRT-PCR, PRL transcripts were below the detection limit at day 0 in both subjects, but became detectable by day 7 (Fig 1F and G). In line with this, epidermal PRL protein IR significantly increased during human skin culture as measured by quantitative immunohistomorphometry (Fig 1H–K). Interestingly, in a third, likely postmenopausal subject, PRL gene expression was detectable at day 0, but significantly decreased in organ culture (data not shown).

### PRLR immunoreactivity and gene expression is present in corporal skin and decreases during organ culture

**Figure 2:**
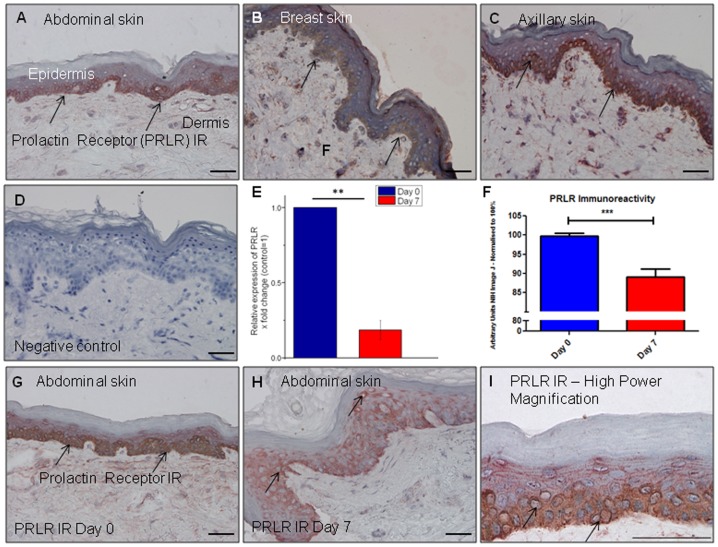
Prolactin receptor immunoreactivity and gene expression is present in corporal skin and decreases during culture. PRL receptor (PRLR) IR is present in the epidermis of corporal from three female subjects aged 28–63 years (A–C). This IR was present in the basal layer of the epidermis (black arrows) in a cytoplasmic distribution. (D) Omission of the primary antibody served as the negative control. (E) PRLR gene expression was detectable in corporal skin from three female subjects (results pooled and expressed as fold change), aged 42–63 years, at day 0 and decreased significantly during serum-free organ culture. PRLR protein expression also significantly decreased during organ culture (F–H). High magnification of PRLR IR shown in (I). Protein expression results were pooled from 3 females aged 42–63 years as in Fig 1(J). Representative photomicrographs of PRLR IR during organ culture are both taken from abdominal skin from 63 year old ♀. All scale bars represent 50 µm.

Next, we determined the expression of PRLR in human corporal by immunohistochemistry. PRLR protein expression was most notable in the basal epidermal layer, again in a cytoplasmic distribution (Fig 2A–C) in each skin site. In contrast to PRL gene expression, PRLR gene expression was detectable in all subjects at day 0, and transcript levels were significantly reduced by day 7 (Fig 2E). This was paralleled by a reduction in PRLR IR between day 0 and 7 (Fig 2F–I). This reciprocal relationship between PRL and PRLR gene expression recapitulates previous observation in murine skin [2].

### Substance P decreases PRL expression in human epidermis

**Figure 3:**
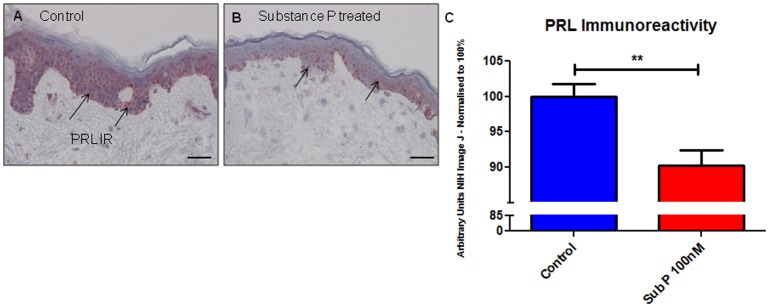
Substance P decreases PRL immunoreactivity in human epidermis. Epidermal PRL IR (black arrows) was examined in full-thickness human skin organ culture. (A) PRL IR in the control group was significantly greater than that in (B) Substance P treated skin. PRL IR was measured using Image J software, showing that (C) epidermal PRL IR was significantly decreased by 100 nM Substance P treatment (p = 0.009). Results were pooled from ♀ subjects aged 45 and 46 years. Scale bars represent 50 µm.

Given that substance P is a recognized potential regulator of pituitary PRL secretion [11] and plays an important role in neurogenic skin inflammation [33], the effect of Substance P on epidermal PRL was determined by quantitative immunohistomorphometry. Substance P treatment decreased PRL IR (black arrows) compared to control skin (Fig 3 A–C).

### Neither CRH nor thyroid hormones regulate PRL expression in human epidermis

As a central hormone in the systemic stress response, which may also regulate pituitary PRL secretion [44], [45], we next investigated the effect of CRH on epidermal PRL expression. CRH had no effect on PRL expression (p = 0.24) (Fig S1A–E). An example of the epidermal area of evaluation is shown in Fig S1A. Similarly, thyroid hormones (T3 and T4) did not alter epidermal PRL or PRLR IR. Furthermore, epidermal PRLR gene expression was not influenced by T3 or T4 over the same time period, when compared to the control skin (Fig S2 A–J).

### IFNγ increases PRL expression in the HF outer root sheath whilst TNFα decreases follicular PRL expression

**Figure 4:**
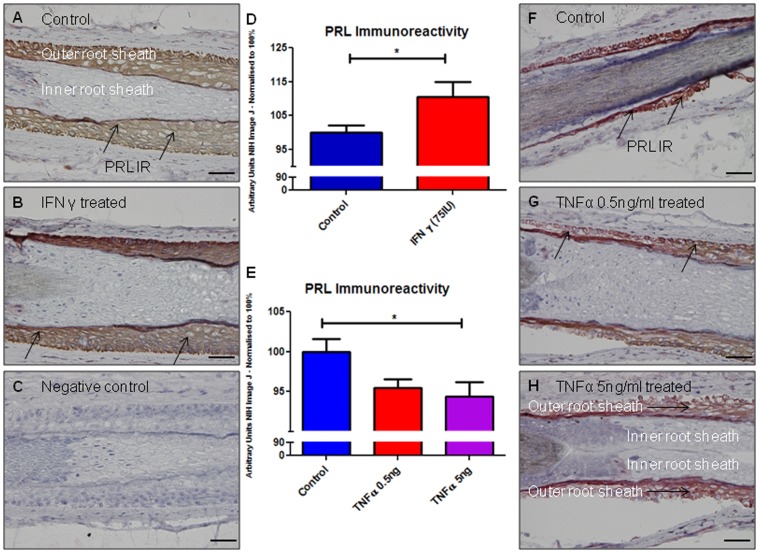
IFNγ increases PRL immunoreactivity in the hair follicle, whilst TNFα decreases follicular PRL immunoreactivity. (A) PRL IR in control hair follicle outer root sheath keratinocytes (black arrows), (B) after INFγ treatment and (C) negative control. (D) PRLR IR is significantly increased by INFγ treatment (p = 0.044). Results were pooled from 3 ♀ subjects (Aged 44–68), 14–16 HFs per group in total. In contrast, TNFα 5 ng/ml significantly decreased PRL IR (E). PRL IR is shown in (F) control hair follicles and those treated with (G) TNFα 0.5 ng/ml and (H) TNFα 5 ng/ml. Results were pooled from 3 ♀ subjects (Aged 49–68) 18–23 HFs per group in total. Scale bars represent 50 µm.

Since pituitary PRL secretion may be regulated by IFNγ and TNFα, albeit in rodent studies [46], [47], [48], [49], and since these cytokines [50], [51] and PRL [3], [5], [28], [52], [53] have been implicated in the pathogenesis of psoriasis, we also examined whether these prototypic pro-inflammatory cytokines influence PRL and PRLR expression in the HF. This examination showed that PRL IR in ORS keratinocytes *in situ* was significantly increased by IFNγ treatment (Fig 4A–D, black arrows). Since 50 ng/ml of TNFα resulted in increased catagen induction (HF regression) (data not shown) [38], resulting in too few anagen HFs for further analysis, only lower TNFα concentrations could be followed up at the protein expression level. This revealed that PRL expression was unaffected by TNFα 0.5 ng/ml, but significantly decreased by 5 ng/ml. (Fig 4E–H).

### Dopamine has no effect on PRL or PRLR IR in the outer root sheath of human HFs at the gene or protein level

Consistent with the conventional wisdom that extra-pituitary PRL production is not under dopaminergic control [18], [54], dopamine treatment of HFs did not alter PRL IR (Fig S3A–E). Indeed, PRLR expression at the gene and protein level (10–1,000 nM), was unaffected by the range of dopamine concentrations tested (Fig S4A–H). A range of dopamine concentrations was tested given that dopamine’s effect on PRL secretion is not linear, and low concentrations have even been found to stimulate PRL release in the lactotrophes *in vitro* [55].

### IFNγ does not alter PRLR gene or protein expression in human HF epithelium

In contrast to its effect on PRL expression, PRLR IR in the outer root sheath of HFs was unchanged by treatment with IFNγ (Fig S5A–D). Also, IFNγ exerted no consistent regulatory effect on PRLR gene expression in the HF (Fig S5E).

### TNFα significantly decreases PRLR IR in the HF epithelium

**Figure 5:**
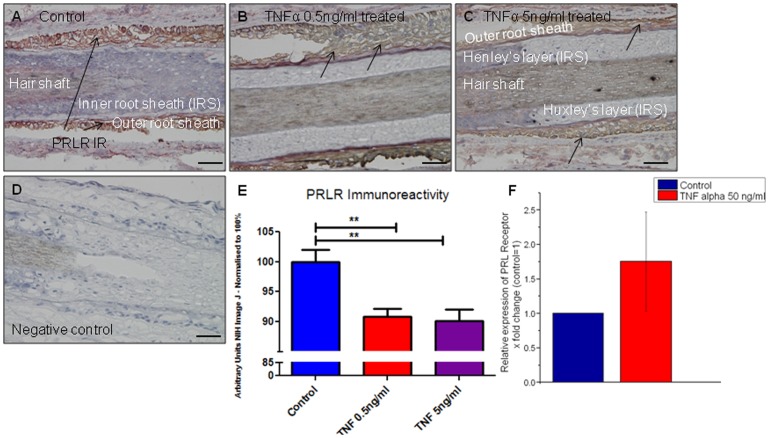
TNFα significantly decreases PRLR immunoreactivity in the outer root sheath of hair follicles. (A) PRLR IR in the outer root sheath of control hair follicles (black arrows) was reduced in comparison PRLR IR after treatment with TNFα at concentrations of (B) 0.5 ng/ml and (C) 5 ng/ml. (D) Negative control. (E) Quantitative analysis confirms decreased PRLR IR after TNFα treatment (13–17 HFs per group in total). Results were pooled from same subjects described in Fig 4. (F) Pooled results of PRLR steady state gene expression in two subjects (♀ aged 53 and 66 years) showed no significant effect of 50 ng TNFα treatment after 48 hours. Scale bars represent 50 µm.

Interestingly, TNFα (0.5–50 ng/ml) significantly decreased PRLR IR in the ORS of human HFs (Fig 5A–E) after 7 days. There was no significant effect of 50 ng/ml TNFα treatment on PRLR gene expression after 48 hours (Fig 5F). For qRT-PCR studies all HFs were used as they could not be histologically staged, whilst for PRLR IR immunohistochemistry only anagen HFs could be analysed.

### TNFα treatment decreases PRL gene expression in cultured ORS keratinocytes

**Figure 6:**
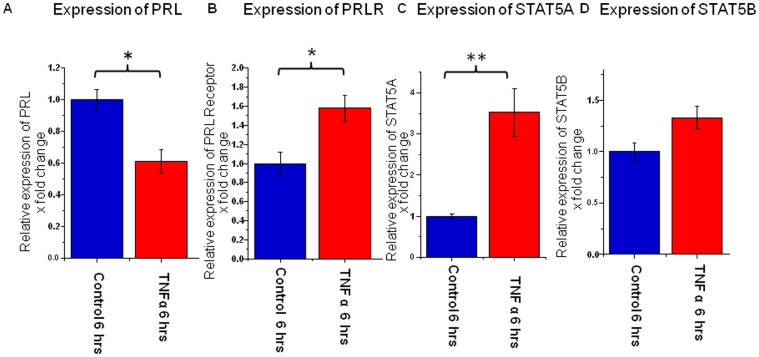
PRL and PRLR are detectable in cultured outer root sheath keratinocytes. (A) PRL and PRLR were detectable in ORS keratinocytes in culture, consistent with the in-situ protein data. TNFα treatment (5 ng/ml) decreased PRL but (B) increased PRLR gene expression. There was also evidence that TNFα modulated STAT5a expression, a key downstream signal of PRLR, but not STAT5b (C–D).

Given that PRL and PRLR IR was prominent in the ORS, we checked PRL and PRLR gene expression in cultured ORS keratinocytes, which expressed PRL and PRLR at the gene level. As in organ-cultured intact human HFs, TNFα 5 ng/ml significantly decreased PRL gene expression in isolated, cultured ORS keratinocytes (Fig 6A). In contrast, TNFα 5 ng/ml increased both PRLR and STAT5a gene expression (whereas levels of STAT5b were not affected) (Fig 6B–D). This shows that the effects of TNFα on PRL gene expression may be independent of the HF mesenchyme.

### PRL increases STAT5 phosphorylation in serum-free organ culture

**Figure 7:**
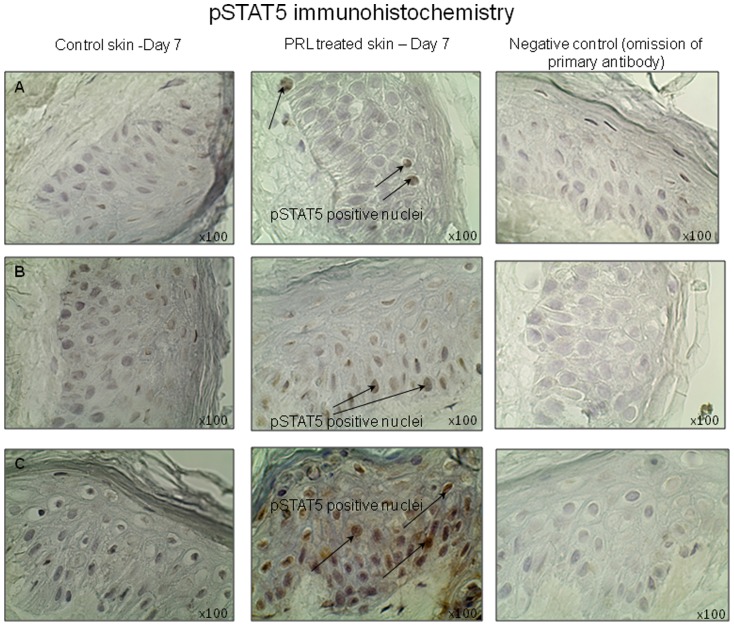
STAT5 phosphorylation is increased by PRL treatment in serum-free skin organ culture. Treatment with PRL (400 ng/ml) in serum-free organ culture increased epidermal STAT5 phosphorylation in all 3 of the individuals investigated. 3 females aged 42–63 years as in Fig 1(J) and 2(I). Arrows show phosphorylated STAT5 as detected by monoclonal rabbit antibody recognising STAT5a and b phosphorylated at Tyr 694.

In order to determine whether the PRLRs detected in human skin are functional, we also examined whether PRL treatment activated epidermal STAT5 phosphorylation. STAT5 is the main mediator of PRLR signalling [56] and is expressed in human epidermis [57]. Indeed, at the end of the culture period, STAT5 phosphorylation was clearly increased in PRL treated skin from three different individuals (Fig. 7A–C), thus indicating functionality of PRLR-mediated signalling in human epidermis.

### The regulation of intracutaneous PRLR expression does not appear to be compartment-specific

Finally, we assessed whether PRL impacts on the intracutaneous expression of its own receptor and whether there are any differences in the regulation of PRLR by its ligand between defined skin compartments. As expected, prominent PRLR IR was seen in the ORS and did not change significantly after PRL treatment on the protein level (Fig S6A–D), Nor did PRL treatment significantly decrease PRLR expression at the gene or protein level in organ-cultured skin (Fig S6E-I). By ELISA, there was no evidence of PRL secretion into the media in either skin organ or HF culture (Fig S6J).

## Discussion

This study demonstrates that contrary to a previous report [9], human non-scalp skin not only expresses PRLR on the gene and protein levels, but also PRL mRNA and protein. That PRL expression increased during human truncal skin organ culture shows that intracutaneous PRL expression occurs even in the absence of vascular, neural, and hypothalamic stimuli. We also demonstrate that PRL and PRLR expression is time-dependent, at least *in vitro*, using a well established immunohistomorphological method for quantifying protein expression, measuring the intensity of protein IR *in situ* [20], [21], [33], [36], [43], [58], [59], [60]. These data from serum-free organ culture demonstrate that human skin can produce PRL, and reconcile apparently conflicting data fom the literature. Fully consistent with Slominski *et al* [9] we were unable to identify PRL gene expression in 2/3 subjects at day 0. However, PRL became detectable during serum free organ culture. It will be intriguing to study whether this temporally regulated PRL gene expression is significant *in vivo,* for example in response to skin trauma or psychological stress.

While we found no evidence that PRL is actively secreted by the skin and/or HFs, PRL secretion into the culture medium cannot be excluded, since it could well have been below the detection limit of the employed ELISA, and may have been detectable with the more sensitive Nb2 cell proliferation bioassay [37].

Importantly, the study identifies the key stress mediator and neurogenic inflammation-associated neuropeptide, substance P, and the pro-inflammatory cytokines, IFNγ and TNFα as regulators of PRL protein expression *in situ* in human truncal skin and HFs. In contrast, dopamine, the classical negative regulator of pituitary PRL secretion, did not exert a significant regulatory effect on PRL expression in human HFs. We deliberately used dopamine itself rather than specific dopamine receptor agonists, for example cabergoline or bromocriptine, as there is no evidence at present that human HFs, or even skin, express the dopamine receptor 2 to which these agents bind. To data, only dopamine receptor 1 transcripts have been demonstrated in human skin and HFs [61]. Unlike the compartment-specific differences that TRH exerts on cutaneous pigmentation [62], there was no evidence that there are skin compartment-specific differences in the regulation of PRLR by its own ligand.

Our group has consistently identified PRL and PRLR expression in female corporal skin from several locations, in a distribution pattern strikingly similar to that seen in scalp skin [20]. However, a recent study has called attention to the substantial variability in the sensitivity and specificity of commercially available PRLR antibodies [63]. Nevertheless, the PRLR staining we report here is consistent with that reported in the literature, which had been using a different PRLR antibody [1]. Due to our existing ethics approval (anonymized samples, with only gender, age, and skin location provided) we are unable to gain information regarding donors’ menopausal status. Given that post-menopausal PRL levels tend to be lower than those in pre-menopausal females [64], possibly as a result of decreased circulating estrogen levels, it is conceivable that the regulation of extra-pituitary PRL expression may also be influenced by menopausal status. However, in other extra-pituitary tissues, for example, glandular breast explants, estradiol did not regulate PRL release [65]. Moreover, the local tissue inflammatory milieu may also influence PRL expression, particularly in the context of psoriasis [28]. Follow-up studies would profit from taking these potential modulators of extrapituitary PRL/PRLR expression into account.

Given that the expression of the pituitary hormone pro-opiomelanocortin (POMC) in murine skin is reportedly up to 10,000 times lower than that in the pituitary [66] highly sensitive methods are required to investigate PRLR expression. Furthermore, another issue to be considered in expression mapping is the generation of isoforms from alternative splicing. This process may prevent detection of some transcripts; well illustrated in the case of CRH receptor type 1[67]. We specifically used qRT-PCR to provide quantitative data on the levels of PRL/PRLR transcript in the organ cultured skin/HFs so as to complement the quantitative protein expression in situ data. However, in follow-up studies, in-situ hybridization data are desirable to confirm the location of PRL/PRLR transcript expression in human skin and HFs, as are Western Blots and fully quantitative ELISA analyses performed on protein extracts, provided the much larger quantities of human tissue that are needed for the latter analyses are available.

To confirm the activity of PRLR-mediated signaling, STAT5 phosphorylation was also investigated at the protein level. In all three subjects tested, PRL treatment increased STAT5 phosphorylation at day 7. This provides preliminary evidence that cutaneous PRLR-mediated signaling can be transduced via the canonical Jak2/STAT5 pathway. However, the extent to which other recognised signaling pathways/proteins may be involved, for example the Ras/Raf/MAPK, and Src kinases or PI3K/Akt pathways, needs to be clarified in future studies. The data also provides indirect evidence that the long form of the PRLR is expressed in the skin as short PRLR isoforms are unable to activate Jak/STAT pathway.

The observed effect of IFNγ on PRL expression did not seem to be related to changes in PRLR expression, since the latter was unchanged. PRL is an important immunomodulator, possibly with overall pro-inflammatory effects in response to the immunosuppressive effects of stress [68]; consistent with the finding that IFNγ increases PRL expression in the HF. In terms of HF pathophysiology, lesional alopecia areata skin has been shown to have increased levels of IFNγ mRNA [69] and IFNγ has potent catagen (HF regression) promoting effects [39]. Intriguingly, PRL can exert the same effects [1], raising the possibility that IFNγ may partially exert its effects via alterations in intrafollicular PRL expression. Future studies utilizing pure PRLR antagonists could address this question [21]. Moreover, PRL also modulate expression of the interferon regulatory factor-1 gene [11], which underscores the potential importance of the “PRL-IFNγ link” in human skin biology and pathology.

In addition to stimulators of PRL and/or PRLR expression we have also identified inhibitors. Substance P decreased PRL IR in the epidermis, whilst TNFα decreased PRL and PRLR IR in the HF. Given that substance P is a key mediator of neurogenic skin inflammation [70], and may partially explain the effects of psychological stress on hair growth in alopecia areata and telogen effluvium [33], it is interesting to note that substance P decreased PRL IR. This is well in line with similar substance P effects reported on pituitary PRL production in rodents [71].

TNFα treatment actually increased PRLR transcription in cultured ORS keratinocytes, in contrast to its effect on PRLR protein expression in the ORS of the intact HF. This raises the question of whether the regulation of PRLR expression in human HF epithelium *in situ* depends on the presence of as yet unidentified HF mesenchyme-derived factors. The down-regulation of follicular PRLR expression by TNFα is also intriguing in view of the importance of TNFα in both inflammatory dermatoses such as psoriasis [51] and hair growth [38]. In follow-up studies, the epidermal regulation of PRL and/or PRLR protein expression by TNF deserves to be characterized, since increased TNFα and PRL levels in psoriasis [28] may both result in a decrease in PRLR expression. The current serum-free human skin organ culture model permits one to investigate whether the regulation of PRL and/or PRLR is altered in inflammatory skin disorders, for example in psoriasis. Also, novel PRLR antagonists [18] can be utilized to determine whether they effectively counteract any cutaneous effects of PRL, for example on keratinocyte proliferation [72] and angiogenesis [73].Moreover, laser-capture microdissection may allow the identification of compartment specific PRL gene expression in the skin, although given that the skin and HF contain immune cells, it may be difficult to determine the contribution they play to intracutaneous PRL production.

It is now appreciated that PRL is an important regulator of human hair growth [1], [6], [21], [74], [75] and may be implicated in the pathobiology of common, chronic skin diseases [6], [27] including psoriasis [3], [4], [5], [28], [52], [76], [77] and lupus erythematosus [7], [78], [79]. Therefore, targeting cutaneous PRL production and PRLR expression via these and other recently identified regulators of intracutaneous PRL and PRLR expression in human skin and its appendages [20] may represent a novel dermatotherapeutic strategy.

## Supporting Information

Figure S1 **CRH does not alter PRL protein expression in human skin.** PRL IR in the control skin (A) was not significantly different to that in (B) CRH treated skin. Positive and negative controls are shown in (C) and (D). Negative control was via omission of the primary antibody and the outer root sheath of the hair follicle and sebaceous gland served as internal positive controls. Quantitative IR measurement, the epidermis, is shown in (A) and evaluation in (E). Results were pooled from 3 ♀ subjects, aged 45–57 years.
(TIF)

Figure S2 **Thyroid hormones have no significant effect on PRL and PRLR expression in human skin**. (A) PRL IR in the epidermis of control skin was not significantly different to that in skin treated with (B) triiodothyronine (T3) or (C) thyroxine (T4) for 24 hours as demonstrated by quantitative measurement of PRL IR (D). Results pooled from 5♀ subjects aged 45–71 years. Similarly, PRLR IR in the epidermis of control skin (E) was not significantly different in (F) T3 or (G) T4 treated skin as quantified in (H). Results pooled from 3♀ subjects aged 56–71 years. Indeed, neither 24 hours of treatment with T3 (I) or T4 (J) significantly affected PRLR gene expression. Results pooled from 2♀ subjects aged 46 and 60 years.
(TIF)

Figure S3 **Dopamine does not alter PRL protein expression in human hair follicles.** PRL IR in control hair follicles (A) was not significantly different to that in Dopamine (1000 nM) treated hair follicles (B). Negative control (C). Quantitative IR measurement revealed no significant differences across the range of Dopamine concentrations tested (D) 1000 nM (pooled results from 3♀ subjects, aged 47–68 years, 21–22 HFs per group in total) and (E) 10–100 nM in 2♀ subjects aged 47–64, 15–21 HFs per group in total). Areas measured by image J are shown in (A).
(TIF)

Figure S4 **Dopamine does not regulate PRLR expression at the gene or protein level in human hair follicles.** PRL IR in the control hair follicles (A) was unchanged compared with that after Dopamine (B) 10 nM, (C) 100 nM or (D) 1000 nM treatment. (E) Negative control. Quantitative IR measurement revealed no significant differences across the range of Dopmaine concentrations tested (F) 1000 nM (pooled results from 3♀ subjects, aged 47–68 years, 20–24 HFs per group in total) and (G) 10–100 nM in 2♀ subjects aged 47–64, 14–18 HFs per group in total). (H) Dopamine 1000 nM exerted no significant effect on steady PRLR gene expression after 48 hours (56 ♀ subject).
(TIF)

Figure S5 **IFNγ has no effect on PRLR immunoreactivity in human hair follicles at the gene or protein level.** (A) PRLR IR in the outer root sheath of control hair follicles (black arrows) was unchanged by treatment with (B) IFNγ 75 IU/ml. (C) Negative control. (D) Quantitative analysis showed no significant difference in PRLR. Results were pooled from 3♀ subjects, aged 44–68 years, 13–16 HFs in total from three patients. **Arbitrary units could not be normalised due to low number of anagen hair follicles. Results were pooled from the same subjects as in Fig 4. (E) There was no evidence that IFNγ influenced PRLR gene expression. Results were pooled from 2♀ aged 53–66 years.
(TIF)

Figure S6 **No evidence of compartment specific regulation of PRLR expression in human skin.** PRLR IR is not significantly reduced by PRL 400 ng on the protein level in the outer root sheath of HFs after serum-free organ culture (A–D). Results pooled from 4 ♀ subjects (aged 49–68 years). 23 HFs per group in total. Moreover, epidermal PRLR IR was also unchanged after organ-culture (E–H). Results pooled from 3 ♀ subjects described in Fig 2. This correlated with no significant difference at the gene transcription level after 7 days in pooled results from three subjects (I) as described in Fig 1. Scale bars represent 50 µm. PRL was not detectable in the conditioned media in either skin or HF organ culture. Level of detectability shown with solid black line (J).
(TIF)

Table S1 **The differential regulation of pituitary and extrapituitary PRL synthesis and release.** There is, at times, a bewildering array of seemingly contradictory regulatory effects on PRL and PRLR expression between depending on the site of PRL production. Some of these are due to methodological factors. Abbreviations: T3:triiodothyronine, T4:thyroxine.
(DOC)
