# Supplementary figures and images for: Tumour Necrosis Factor Alpha, Interferon Gamma and Substance P Are Novel Modulators of Extrapituitary Prolactin Expression in Human Skin
Source: PLoS One. 2013 Apr 23;8(4):e60819. doi: 10.1371/journal.pone.0060819 (PMC3634033; doi:10.1371/journal.pone.0060819)

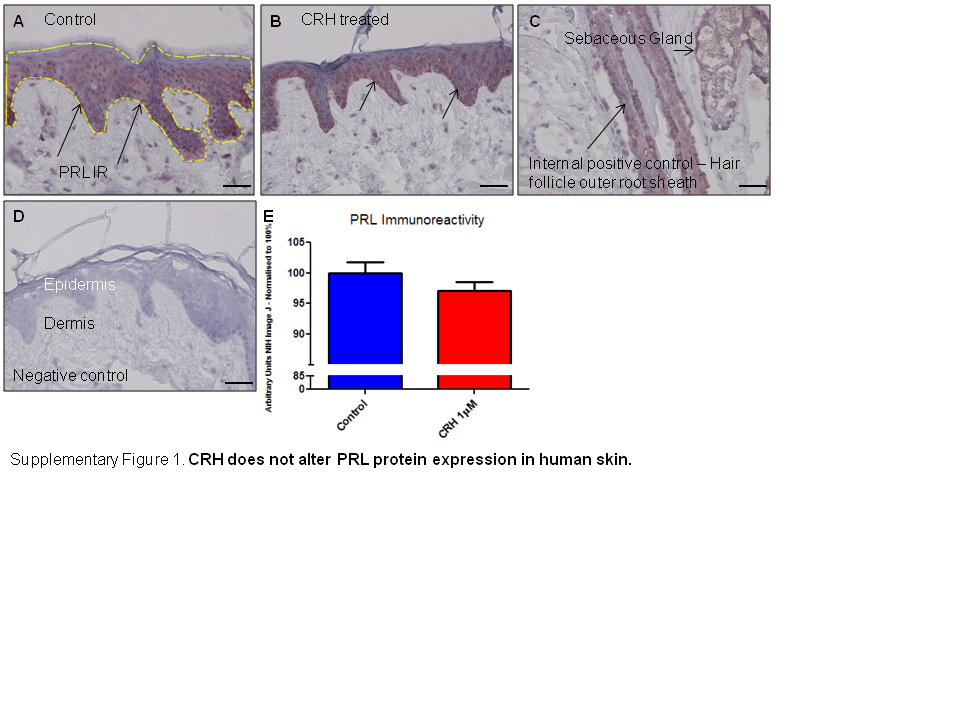

Supplement: Figure S1 — CRH does not alter PRL protein expression in human skin. PRL IR in the control skin (A) was not significantly different to that in (B) CRH treated skin. Positive and negative controls are shown in (C) and (D). Negative control was via omission of the primary antibody and the outer root sheath of the hair follicle and sebaceous gland served as internal positive controls. Quantitative IR measurement, the epidermis, is shown in (A) and evaluation in (E). Results were pooled from 3 ♀ subjects, aged 45–57 years. (TIF) [file pone.0060819.s001.tif]

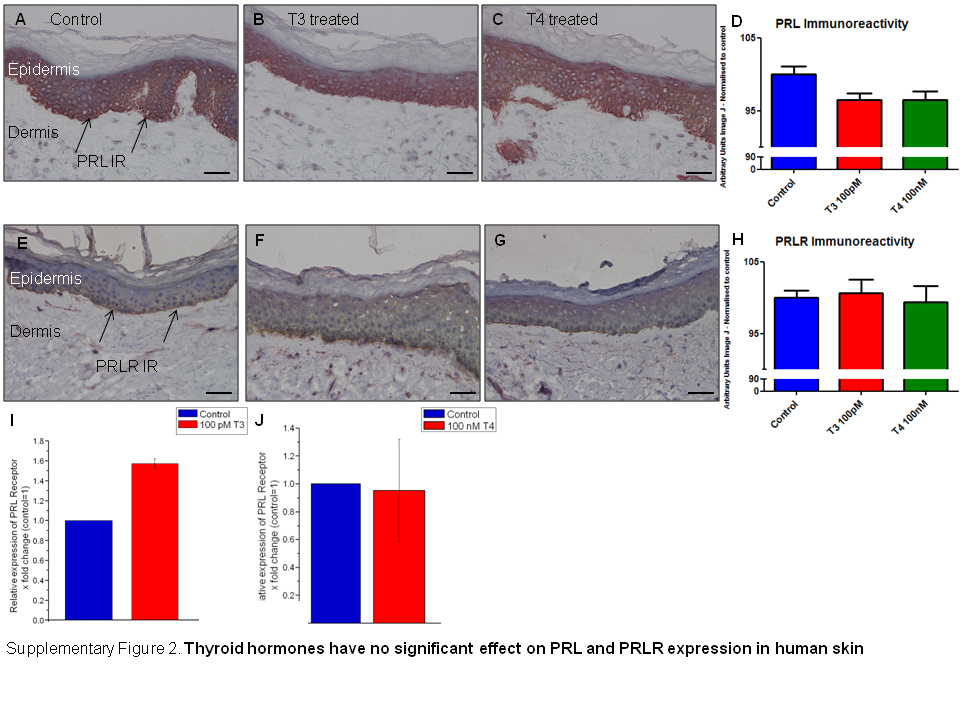

Supplement: Figure S2 — Thyroid hormones have no significant effect on PRL and PRLR expression in human skin. (A) PRL IR in the epidermis of control skin was not significantly different to that in skin treated with (B) triiodothyronine (T3) or (C) thyroxine (T4) for 24 hours as demonstrated by quantitative measurement of PRL IR (D). Results pooled from 5♀ subjects aged 45–71 years. Similarly, PRLR IR in the epidermis of control skin (E) was not significantly different in (F) T3 or (G) T4 treated skin as quantified in (H). Results pooled from 3♀ subjects aged 56–71 years. Indeed, neither 24 hours of treatment with T3 (I) or T4 (J) significantly affected PRLR gene expression. Results pooled from 2♀ subjects aged 46 and 60 years. (TIF) [file pone.0060819.s002.tif]

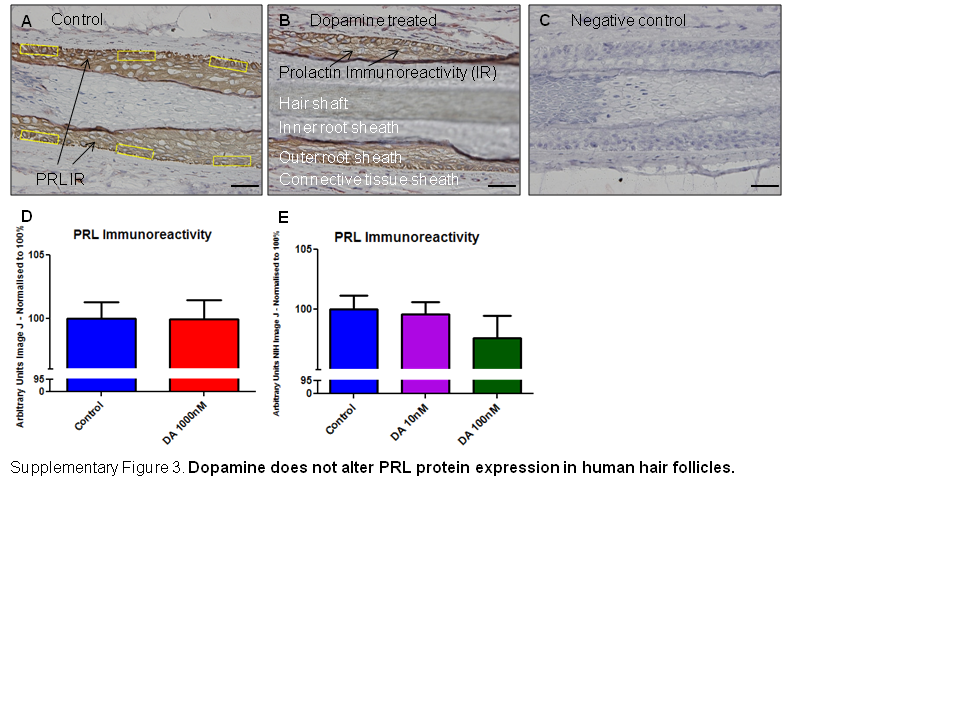

Supplement: Figure S3 — Dopamine does not alter PRL protein expression in human hair follicles. PRL IR in control hair follicles (A) was not significantly different to that in Dopamine (1000 nM) treated hair follicles (B). Negative control (C). Quantitative IR measurement revealed no significant differences across the range of Dopamine concentrations tested (D) 1000 nM (pooled results from 3♀ subjects, aged 47–68 years, 21–22 HFs per group in total) and (E) 10–100 nM in 2♀ subjects aged 47–64, 15–21 HFs per group in total). Areas measured by image J are shown in (A). (TIF) [file pone.0060819.s003.tif]

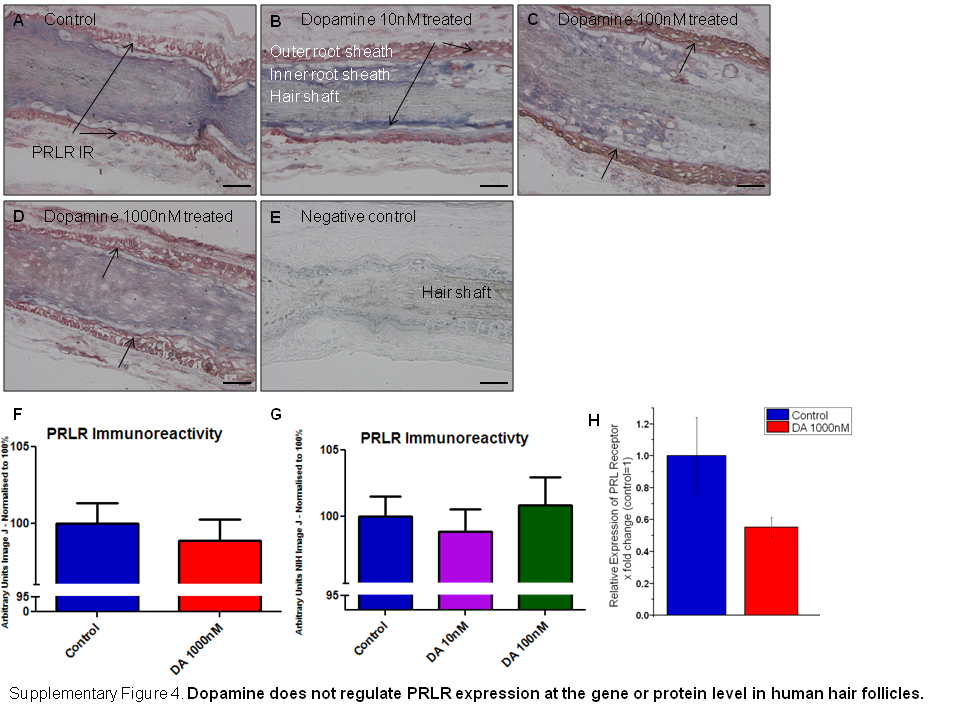

Supplement: Figure S4 — Dopamine does not regulate PRLR expression at the gene or protein level in human hair follicles. PRL IR in the control hair follicles (A) was unchanged compared with that after Dopamine (B) 10 nM, (C) 100 nM or (D) 1000 nM treatment. (E) Negative control. Quantitative IR measurement revealed no significant differences across the range of Dopmaine concentrations tested (F) 1000 nM (pooled results from 3♀ subjects, aged 47–68 years, 20–24 HFs per group in total) and (G) 10–100 nM in 2♀ subjects aged 47–64, 14–18 HFs per group in total). (H) Dopamine 1000 nM exerted no significant effect on steady PRLR gene expression after 48 hours (56 ♀ subject). (TIF) [file pone.0060819.s004.tif]

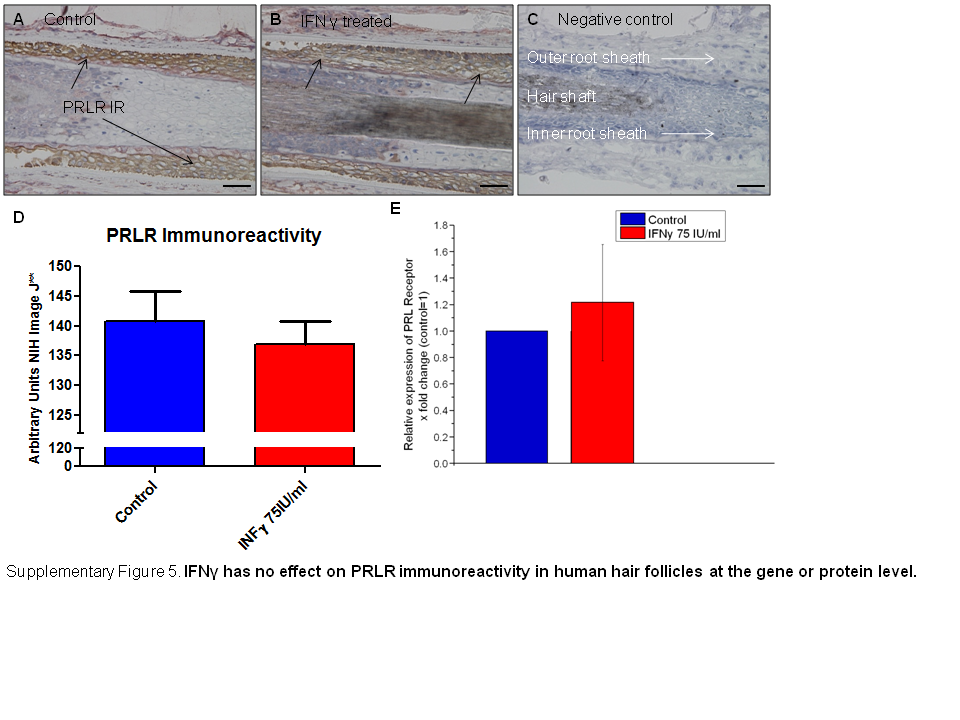

Supplement: Figure S5 — IFNγ has no effect on PRLR immunoreactivity in human hair follicles at the gene or protein level. (A) PRLR IR in the outer root sheath of control hair follicles (black arrows) was unchanged by treatment with (B) IFNγ 75 IU/ml. (C) Negative control. (D) Quantitative analysis showed no significant difference in PRLR. Results were pooled from 3♀ subjects, aged 44–68 years, 13–16 HFs in total from three patients. **Arbitrary units could not be normalised due to low number of anagen hair follicles. Results were pooled from the same subjects as in Fig 4. (E) There was no evidence that IFNγ influenced PRLR gene expression. Results were pooled from 2♀ aged 53–66 years. (TIF) [file pone.0060819.s005.tif]

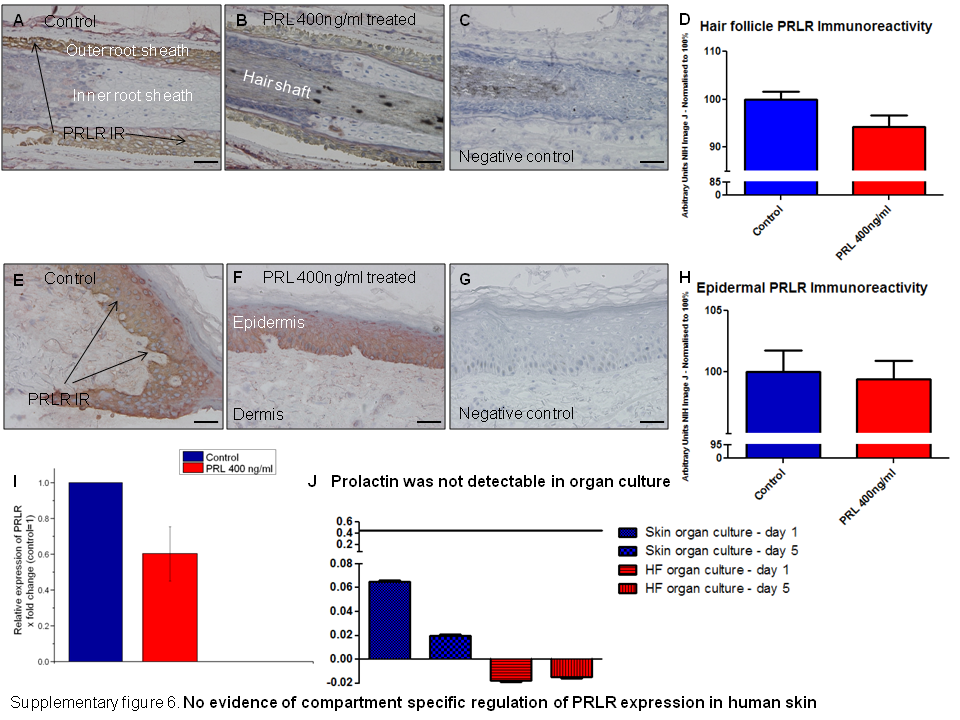

Supplement: Figure S6 — No evidence of compartment specific regulation of PRLR expression in human skin. PRLR IR is not significantly reduced by PRL 400 ng on the protein level in the outer root sheath of HFs after serum-free organ culture (A–D). Results pooled from 4 ♀ subjects (aged 49–68 years). 23 HFs per group in total. Moreover, epidermal PRLR IR was also unchanged after organ-culture (E–H). Results pooled from 3 ♀ subjects described in Fig 2. This correlated with no significant difference at the gene transcription level after 7 days in pooled results from three subjects (I) as described in Fig 1. Scale bars represent 50 µm. PRL was not detectable in the conditioned media in either skin or HF organ culture. Level of detectability shown with solid black line (J). (TIF) [file pone.0060819.s006.tif]
