# Supplementary material for: Tumour Necrosis Factor Alpha, Interferon Gamma and Substance P Are Novel Modulators of Extrapituitary Prolactin Expression in Human Skin
Source: PLoS One. 2013 Apr 23;8(4):e60819. doi: 10.1371/journal.pone.0060819 (PMC3634033; doi:10.1371/journal.pone.0060819)
Supplement: Table S1 — The differential regulation of pituitary and extrapituitary PRL synthesis and release. There is, at times, a bewildering array of seemingly contradictory regulatory effects on PRL and PRLR expression between depending on the site of PRL production. Some of these are due to methodological factors. Abbreviations: T3:triiodothyronine, T4:thyroxine. (DOC) [file pone.0060819.s007.doc]

***Supplementary information table.***

| **Regulator** | **Effect on Pituitary PRL** | **Reference** | **Effect on Extrapituitary PRL** | **Reference** |
| --- | --- | --- | --- | --- |
| **Bromocriptine** | Potent inhibitor - via binding of dopamine (DA) receptor and degradation of PRL in cultured anterior pituitary cells | [1] | No effect on PRL secretion in decidual explants  No effect on level of PRL in amniotic fluid | [2]  [3] |
| **Corticotropin-releasing hormone** | Increased serum PRL in rats *in vivo*, but no effect alone on pituitary cell PRL secretion *in vitro*  Ovine CRH induces increased PRL in petrosal sinus blood in patients with Cushing’ Syndrome | [4]  [5] | Increased decidualisation in human endometrial stromal cells, with increased PRL in media | [6] |
| **Dopamine** | Potent inhibitor of PRL release – via binding of DA 2 Receptor | [7] | No effect of PRL secretion in decidual explants  No effect on synthesis or release of decidual glycosylated PRL *in vitro* | [2]  [8] |
| **Estrogens** | Stimulation of synthesis and release of pituitary PRL | [7] | Increased PRL immunoreactivity in human skin | [9] |
| **Insulin** | Increased PRL gene expression in rat derived GH_3_ cell lines | [10] | Stimulation of synthesis and release from human decidual cells | [11] |
| **Insulin-like growth factor** | Increased PRL secretion in cells derived from human prolactinomas | [12] | Increased PRL in medium from decidualised human stromal cell cultures, but decreased PRL at higher concentrations | [13] |
| **Interferon gamma** | Attenuated basal and induced PRL secretion from rat pituitary cell lines  Increased PRL secretion in primary rat pituitary cell cultures  Increased serum PRL levels after intraperitoneal administration in male rats | [14]  [15]  [16] | Inhibition of PRL gene expression in endometrial stromal cells | [17] |
| **Substance P** | Conflicting reports in the literature. Substance P can stimulate and inhibit PRL secretion depending on dose and method of administration. | [18,19] |  |  |
| **T3/T4** | T3 induces inhibition of PRL signalling via STAT 5a and b in a human embryonic fibroblast cell line | [20] | T4 increases PRLR in rat prostate | [21] |
| **Thyrotropin releasing hormone** | Stimulation of PRL secretion from rat pituitary tumour cells. | [22] | No effect of PRL secretion in decidual explants  Increased PRL expression in cultured human hair follicles | [2]  [9] |
| **Tumour necrosis factor alpha** | Suppression of PRL release and inhibition of response to TRH in cultured rat anterior pituitary cells  Stimulation of PRL release from rat pituitary cells | [23]  [24] | Inhibition of the synthesis and release of decidual PRL | [25] |

**The differential regulation of pituitary and extrapituitary PRL synthesis and release.** There is, at times, a bewildering array of seemingly contradictory regulatory effects on PRL and PRLR expression between depending on the site of PRL production. Some of these are due to methodological factors. Abbreviations: T3:triiodothyronine, T4:thyroxine.

1. Ben-Jonathan N, Arbogast LA, Hyde JF (1989) Neuroendocrine [corrected] regulation of prolactin release. Prog Neurobiol 33: 399-447.

2. Golander A, Barrett J, Hurley T, Barry S, Handwerger S (1979) Failure of bromocriptine, dopamine, and thyrotropin-releasing hormone to affect prolactin secretion by human decidual tissue in vitro. J Clin Endocrinol Metab 49: 787-789.

3. Lehtovirta P, Ranta T (1981) Effect of short-term bromocriptine treatment on amniotic fluid prolactin concentration in the first half of pregnancy. Acta Endocrinol (Copenh) 97: 559-561.

4. Morel G, Enjalbert A, Proulx L, Pelletier G, Barden N, et al. (1989) Effect of corticotropin-releasing factor on the release and synthesis of prolactin. Neuroendocrinology 49: 669-675.

5. Loli P, Boccardi E, Branca V, Bramerio M, Barberis M, et al. (1998) Growth hormone and prolactin responses to corticotrophin-releasing-hormone in patients with Cushing's disease: a paracrine action of the adenomatous corticotrophic cells? Clin Endocrinol (Oxf) 49: 433-439.

6. Ferrari A, Petraglia F, Gurpide E (1995) Corticotropin releasing factor decidualizes human endometrial stromal cells in vitro. Interaction with progestin. J Steroid Biochem Mol Biol 54: 251-255.

7. Ben-Jonathan N, Mershon JL, Allen DL, Steinmetz RW (1996) Extrapituitary prolactin: distribution, regulation, functions, and clinical aspects. Endocr Rev 17: 639-669.

8. Lee DW, Markoff E (1986) Synthesis and release of glycosylated prolactin by human decidua in vitro. J Clin Endocrinol Metab 62: 990-994.

9. Langan EA, Ramot Y, Hanning A, Poeggeler B, Biro T, et al. (2010) Thyrotropin-releasing hormone and oestrogen differentially regulate prolactin and prolactin receptor expression in female human skin and hair follicles in vitro. Br J Dermatol 162: 1127-1131.

10. Stanley F (1988) Stimulation of prolactin gene expression by insulin. J Biol Chem 263: 13444-13448.

11. Thrailkill KM, Golander A, Underwood LE, Richards RG, Handwerger S (1989) Insulin stimulates the synthesis and release of prolactin from human decidual cells. Endocrinology 124: 3010-3014.

12. Atkin SL, Landolt AM, Foy P, Jeffreys RV, Hipkin L, et al. (1994) Effects of insulin-like growth factor-I on growth hormone and prolactin secretion and cell proliferation of human somatotrophinomas and prolactinomas in vitro. Clin Endocrinol (Oxf) 41: 503-509.

13. Irwin JC, de las Fuentes L, Dsupin BA, Giudice LC (1993) Insulin-like growth factor regulation of human endometrial stromal cell function: coordinate effects on insulin-like growth factor binding protein-1, cell proliferation and prolactin secretion. Regul Pept 48: 165-177.

14. Vankelecom H, Matthys P, Denef C (1997) Involvement of nitric oxide in the interferon-gamma-induced inhibition of growth hormone and prolactin secretion in anterior pituitary cell cultures. Mol Cell Endocrinol 129: 157-167.

15. Yamaguchi M, Koike K, Matsuzaki N, Yoshimoto Y, Taniguchi T, et al. (1991) The interferon family stimulates the secretions of prolactin and interleukin-6 by the pituitary gland in vitro. J Endocrinol Invest 14: 457-461.

16. Cano P, Cardinali DP, Jimenez V, Alvarez MP, Cutrera RA, et al. (2005) Effect of interferon-gamma treatment on 24-hour variations in plasma ACTH, growth hormone, prolactin, luteinizing hormone and follicle-stimulating hormone of male rats. Neuroimmunomodulation 12: 146-151.

17. Christian M, Marangos P, Mak I, McVey J, Barker F, et al. (2001) Interferon-gamma modulates prolactin and tissue factor expression in differentiating human endometrial stromal cells. Endocrinology 142: 3142-3151.

18. Arisawa M, Snyder GD, Yu WH, De Palatis LR, Ho RH, et al. (1990) Physiologically significant inhibitory hypothalamic action of substance P on prolactin release in the male rat. Neuroendocrinology 52: 22-27.

19. Freeman ME, Kanyicska B, Lerant A, Nagy G (2000) Prolactin: structure, function, and regulation of secretion. Physiol Rev 80: 1523-1631.

20. Favre-Young H, Dif F, Roussille F, Demeneix BA, Kelly PA, et al. (2000) Cross-talk between signal transducer and activator of transcription (Stat5) and thyroid hormone receptor-beta 1 (TRbeta1) signaling pathways. Mol Endocrinol 14: 1411-1424.

21. Tiong TS, Stevenson JL, Herington AC (1992) Regulation of prolactin receptor gene expression by thyroid hormone status in the rat. J Mol Endocrinol 8: 63-72.

22. Aizawa T, Hinkle PM (1985) Thyrotropin-releasing hormone rapidly stimulates a biphasic secretion of prolactin and growth hormone in GH4C1 rat pituitary tumor cells. Endocrinology 116: 73-82.

23. Harel G, Shamoun DS, Kane JP, Magner JA, Szabo M (1995) Prolonged effects of tumor necrosis factor-alpha on anterior pituitary hormone release. Peptides 16: 641-645.

24. Yamaguchi M, Koike K, Yoshimoto Y, Ikegami H, Miyake A, et al. (1991) Effect of TNF-alpha on prolactin secretion from rat anterior pituitary and dopamine release from the hypothalamus: comparison with the effect of interleukin-1 beta. Endocrinol Jpn 38: 357-361.

25. Jikihara H, Handwerger S (1994) Tumor necrosis factor-alpha inhibits the synthesis and release of human decidual prolactin. Endocrinology 134: 353-357.
